# A Case Report on a Challenging Infection: Pyogenic Flexor Tenosynovitis

**DOI:** 10.7759/cureus.79785

**Published:** 2025-02-27

**Authors:** Margarida Gomes, Diana Pedrosa, Marcio Oliveira, Rosana Pinheiro, Diogo Barros

**Affiliations:** 1 Orthopaedics and Traumatology, Unidade Local de Saúde do Nordeste, Macedo de Cavaleiros, PRT; 2 Orthopaedic Surgery, Unidade Local de Saúde do Nordeste, Macedo de Cavaleiros, PRT

**Keywords:** antibiotic therapy, hand infections, kanavel signs, pyogenic tenosynovitis, urgent surgical drainage

## Abstract

Pyogenic flexor tenosynovitis is an infection of the flexor tendon sheaths that represents 2.5-9.4% of all hand infections. It is most commonly caused by penetrating trauma and remains a challenging problem, mostly due to the difficult diagnoses, which can lead to delays in treatment and devastating outcomes.

We present a case of a 64-year-old female patient who presented to the Emergency Department with pain and swelling of the fingers of the left hand. Urgent surgical drainage was performed. Intraoperatively, all flexor tendon sheaths were found to be affected. After surgery, she completed one month of oral antibiotherapy and underwent intensive rehabilitation. She recovered hand mobility and sensitivity completely.

Pyogenic flexor tenosynovitis is a devastating infection where outcomes depend on prompt and timely recognition and appropriate treatment. More studies are needed to find new ways to improve the preoperative diagnosis of PFT and new treatment algorithms.

## Introduction

Pyogenic flexor tenosynovitis (PFT) or septic/suppurative flexor tenosynovitis, represents 2.5-9.4% of all hand infections [1]. This closed-space infection of the flexor tendon sheaths of the hand can be caused by local inoculation (wounds, bites) or, less commonly, via hematogenous spread [2,3].

The most common microbiological agents are skin flora (75% of cases with positive cultures for Staphylococcus (S.) aureus; less frequent organisms are S. epidermidis, β-hemolytic streptococci, and Pseudomonas aeruginosa) [3,4].

This type of infection remains a challenging problem, mostly due to the difficulty in diagnosing, which can lead to delays in treatment and devastating outcomes such as motion impairment, deformities, or amputation.

Kanavel signs (exquisite tenderness over the course of the sheath; resting flexed posture of the finger; pain on extending the finger; and fusiform swelling of the whole finger) [2] can be helpful in the classification of PFT, thus helping identify preoperative risk factors associated with worse outcomes and risk of amputation.

The literature is not uniform regarding the treatment of PFT. The gold standard is irrigation and debridement of the flexor sheath associated with antibiotic treatment. In some cases (early diagnosis, absence of abscess or sepsis) empirical IV antibiotics, high arm elevation, and splinting can be considered alternatives to surgical debridement [1,3].

Even with timely adequate treatment and a successful eradication of the infection, some patients continue experiencing pain, swelling, stiffness, loss of mobility, and compromised function of the hand, or even recurrence, which can potentially lead to an amputation (the complication rate is as high as 38%) [2,5].

We present an uncommon case of pyogenic flexor tenosynovitis without a history of trauma, with the presence of pus in the flexor tendon sheaths of all fingers.

## Case presentation

**Figure 1:**
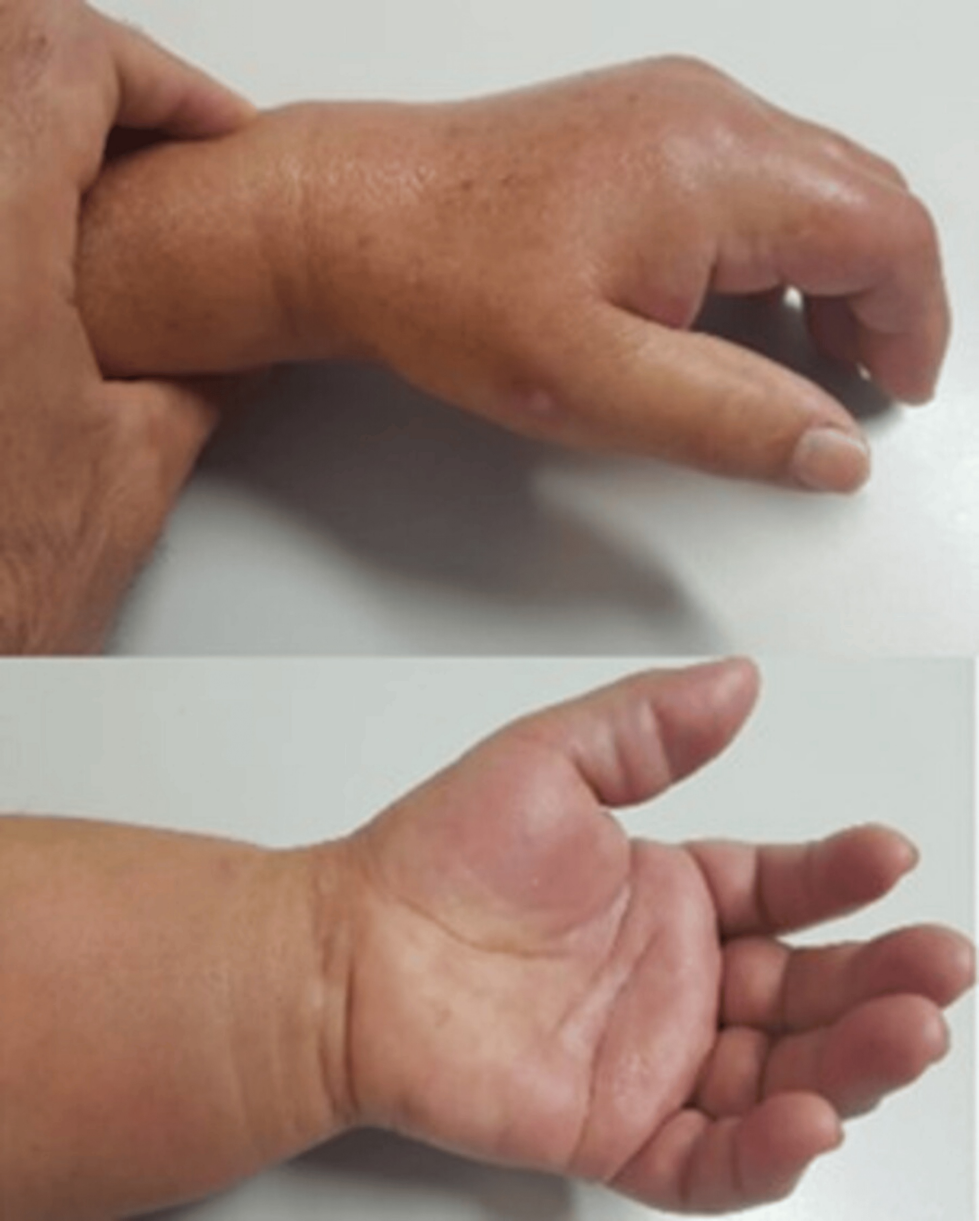
Clinical appearance of the patient's left hand Swelling and redness of the left hand

A 64-year-old female, with no relevant medical history, presented to the Emergency Department with pain and swelling of the fingers of the left hand (Figure 1) and 48 hours of evolution. She denied a history of previous trauma. In the last six hours, she had experienced worsening pain (VAS 10/10), aggravated when attempting to extend the fingers. She presented with fingers in passive flexion, fusiform digital edema, and paresthesia in the distribution of the median nerve. The main point of interest of this particular case relates to the fact that its causative mechanism was not trauma and that it affected the flexor tendons of all fingers (whereas the majority of the cases described in the literature had only one or two fingers affected).

Table 1 shows the laboratory results. Analytically, leukocytosis, neutrophilia, high CRP, and high ESR were observed.

**Table 1:** Laboratory results Leukocytosis (14500 x 10^9/L), neutrophilia (85%), and raised CRP (13.11 mg/dl) and ESR (44 mm/hr) were observed. CRP: C-reactive protein; ESR: erythrocyte sedimentation rate

Parameter	Results	Reference Value
Leukocytes	14500 x 10^9/L	3 - 11 x 10^9/L
Neutrophils	85%	45 - 70%
CRP	13.11 mg/dL	< 1.0 mg/dL
ESR	44 mm/hr	< 20 mm/h

**Figure 2:**
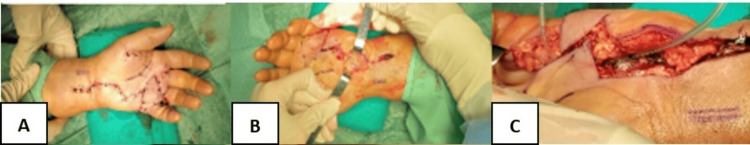
A: Planned incision; B: Z approach through the fourth and fifth palmar rays; C: Appearance after completing debridement and placing a surgical drain

**Figure 3:**
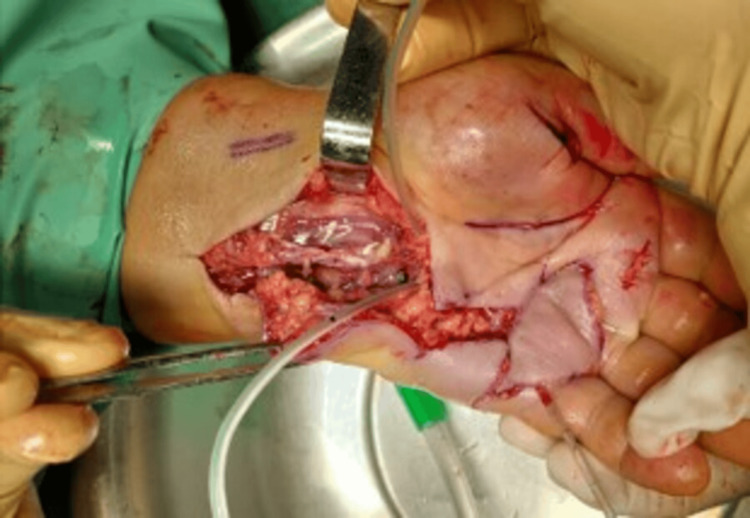
View of the carpal tunnel after incision and debridement

A provisional diagnosis of pyogenic flexor tenosynovitis was made, and urgent surgical treatment was decided upon: A Z approach was adopted through the fourth and fifth palmar rays to the carpus (Figure 2). After incising the transverse carpal ligament, there was drainage of pus and some areas of damage were observed in the median nerve (Figure 3). The flexor sheath was opened from D1 to D5 and a catheter was passed along the sheath (Figure 2). Five samples were collected for microbiology.

After surgery, an empirical antibiotic therapy with vancomycin (IV) was started. This was later adjusted to oral amoxicillin-clavulanate, as culture results revealed multi-sensitive (MS) Staphylococcus aureus (sensitive to amoxicillin, tetracycline, gentamicin, levofloxacin, co-trimoxazole, and teicoplanin, and resistant to penicillin).

After one week of hospital stays the patient experienced positive clinical evolution, resolution of clinical inflammatory signs, decrease in inflammatory markers (leukocytosis of 7000 x 10^9/L; CRP of 3 mg/dl; ESR of 31 mm/hr), resolution of pain (visual analog scale (VAS) 1), improvement of finger mobility, and paresthesia of the median nerve.

**Figure 4:**
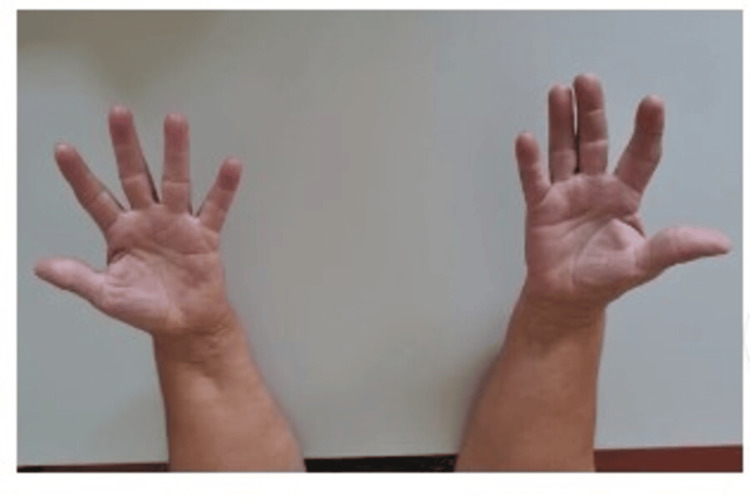
Clinical result one year after surgery with complete range of motion of the fingers

The patient completed one month of oral antibiotherapy and underwent intensive rehabilitation. There was periodic follow-up in the outpatient department, and at one year, finger mobility and sensitivity were recovered and symmetrical to contralateral (Figure 4); VAS at this point was 0.

## Discussion

Pyogenic flexor tenosynovitis is a devastating infection within a closed space that constitutes an orthopedic emergency, as outcomes depend on prompt and timely recognition and appropriate treatment.

Clinically, PFT can be similar to other finger infections, with the presence of pain, redness, and functional limitation. In 1912, Dr. Allen B. Kanavel described four cardinal signs that are still used to this day to diagnose PFT: exquisite tenderness over the course of the sheath; resting flexed posture of the finger; pain on extending the finger; and fusiform swelling of the whole finger [2]. Known as the Kanavel signs, these have high sensitivity for detecting PFT and remain the most useful diagnostic tool for PFT, although their specificity on an individual basis is limited, and only about 54% of patients demonstrate all 4 signs [6]. 

Kennedy et al. found that tenderness to the flexor tendon sheath and pain with passive extension are independent predictors of PFT [6]. Flexion posture and fusiform swelling are also frequently present in patients with PFT but are not sufficiently independent to differentiate PFT from other infections (low specificity) [6]. On the other hand, fusiform swelling is the most sensitive of Kanavel’s signs and is present in 97% of PFT cases [6]. Pain on passive extension of the digit is most commonly the first Kanavel sign to manifest, while flexor sheath tenderness is usually the last to be detected [1].

In this case, all four Kanavel cardinal signs were present, which contributed greatly to our ability to diagnose PFT promptly.

Along with the clinical findings, a hand X-ray should be also obtained to rule out the retention of a foreign body. Hand ultrasonography (US) may be useful for visualizing the flexor tendon and detecting the presence of fluid collection within the flexor sheath, however, it does not differentiate pus from blood. Magnetic resonance imaging (MRI) is not normally used, as it cannot differentiate PFT from other inflammatory conditions, although it can identify the extent of the infection [1].

Although not specific to PFT, inflammatory markers are usually elevated and can be important to monitor the infection and response to treatment [1].

The causative microorganism in this case was Methicilin-sensitive Staphylococcus aureus, the most frequently responsible agent in the literature [2,5,7].

The literature is not uniform, regarding the treatment of PFT. Conservative treatment with empirical IV antibiotics, high arm elevation, and splinting can be considered as an alternative to surgical debridement in patients with PFT who present early (24-48 h of evolution of symptoms), without sepsis or an abscess [1,3]. The time limit or the criteria that define the success or failure of conservative treatment are not yet defined.

In most cases, antibiotics in combination with surgery are the mainstay of treatment. Numerous surgical approaches and techniques can be used to decompress and irrigate the flexor tendon sheath and treat PFT, but there is no consensus regarding the optimal timing and type of intervention. Based on intraoperative findings, Micho et al. developed a three-tiered staging system for PFT: first stage - increased serous exudative fluid; second stage - purulent fluid; third stage - septic necrosis [2].

PFT presenting in stages 1 and 2 could be treated through a small incision, drainage, and irrigation (closed flexor sheath catheter irrigation): a proximal incision made over the level of the A1 pulley and a distal incision proximal to the distal interphalangeal crease [8]. Tissues are then dissected and the flexor tendon sheath is identified. A small incision is made through the A1 pulley, and samples from the fluid inside the flexor sheath are collected for microbiological examination. A second incision is made in the sheath at the A5 pulley, followed by passing a 16/18-gauge angiocatheter or cannula through one of the two incisions and injecting normal sterile saline from the catheter [2,9]. This appears to have better results (especially less finger stiffness and tendon adhesions) when compared to the open technique [9].

Stage 3 PFT should be treated with open debridement made through a volar incision (a straight mid-axial incision or a Bruner zigzag incision) that exposes the flexor sheath. Flexor sheath A1 and A5 pulleys are then incised, the sheath is debrided and washout is done with normal sterile saline [2,7,9]. If the extent and exposure are too small to clear the infection, the two initial incisions can be connected. Pulleys from A2 to A4 should be preserved to avoid tendon bowstringing.

The irrigation fluid is normal saline, as systematic reviews reported that the use of antibiotics had no clear benefit [5]. More recently there have been works with local injection of antibiotics and corticosteroids into the tendon sheath [10]. Local antibiotics may be helpful in limiting bacterial counts and biofilm formation, and corticosteroids may decrease finger stiffness associated with PFT.

Antibiotics can be initiated only after the surgical debridement or as soon as the diagnosis is made, as there is no apparent difference in terms of outcomes [5]. The duration of antibiotic therapy is dependent on clinical evolution (in most cases, two to six weeks after surgery).

Better functional results are obtained when treatment (sheath drainage and antibiotic therapy) is initiated in the first 48 to 72 hours [2,4].

In this case, we performed an urgent surgical approach within 48 hours after the beginning of the symptoms. The presence of pus in the sheath of all flexors is rare and motivated the wider open approach in this case.

The main point of interest of this particular case relates to the fact that its causative mechanism was not trauma and that it affected the flexor tendons of all rays (whereas the majority of the cases described in the literature had only one or two rays affected).

## Conclusions

A high index of suspicion for pyogenic flexor tenosynovitis is essential for early recognition of this diagnosis, which is needed to minimize the devastating consequences associated with treatment delays. This type of infection remains a challenging problem, mainly due to delays in diagnosis and, consequently, treatment. Kanavel signs are a sensitive tool for evaluating a patient with potential PFT but have limited specificity. Along with clinical findings, hand X-ray and ultrasonography may also be useful.

In the future, it is crucial to find new ways to improve the preoperative diagnosis of PFT and investigate and test new treatment algorithms such as conservative treatment with systemic antibiotics without surgical intervention and local antibiotics plus corticosteroids.
